# Multi-Omics Reveals Protected Cultivation Improves Chinese Plum (*Prunus salicina* L.) Quality via Light-Regulated Sugar Metabolism

**DOI:** 10.3390/plants15010164

**Published:** 2026-01-05

**Authors:** Liangliang Cao, Xi Long, Xiaolou Zhu, Jiangong Wang, Weidong Xu, Qiang Lu, Zanyu Ruan, Jiashun Miao, Zhangliang Yao

**Affiliations:** 1 Jiaxing Academy of Agricultural Science, Jiaxing 314016, China; 15706739259@163.com (L.C.); 18357330250@163.com (J.W.); xudong.human@163.com (W.X.); jxluqiang@163.com (Q.L.); 2 Tongxiang Institute of Agricultural Science, Jiaxing Academy of Agricultural Science, Tongxiang 314500, China; 3 College of Agriculture and Biotechnology, Zhejiang University, Hangzhou 310058, China; longxi20030712@163.com; 4 Tongxiang City Forestry Work Station, Tongxiang 314500, China; zxl_260@126.com; 5 Haining Soil and Fertilizer Plant Protection Energy Technology Service Station, Haining 314000, China; 6 Xianghu Laboratory, Hangzhou 310021, China

**Keywords:** *Prunus salicina*, protected cultivation, light regulation, fruit quality, multi-omics analysis

## Abstract

The Chinese plum (*Prunus salicina* L.), ‘Zuili’, is a geographically protected cultivar that is valued for its high polyphenol levels and distinctive flavor. Light availability strongly influences sugar accumulation and secondary metabolism in plum fruit, yet the molecular processes associated with quality variation under protected cultivation remain unclear. Here, we compare three cultivation systems—multi-span greenhouse (M), retractable electric rain shelter (R), and conventional open field (CK)—to evaluate their effect on fruit quality using integrated transcriptomic and metabolomic analyses. Field trials showed that M treatment increased fruit sweetness by 28.10% versus CK (14.68 vs. 11.46 °Brix, *p* < 0.001) without yield loss and significantly improved vertical fruit diameter. RNA-seq analysis identified 7561 and 7962 upregulated genes in the M and R treatments compared to CK, respectively, with significant functional enrichment in pathways related to sucrose metabolism, light-response, and ethylene-mediated signaling. Untargeted metabolomic signaling identified 1373 metabolites, with shading treatments increasing the abundance of several sugar-conjugated compounds (e.g., epicatechin 3-O-(2-trans-cinnamoyl)-β-D-allopyranoside). Multi-omics integration revealed coordinated changes in gene expression and metabolite abundance, suggesting that controlled light environments are associated with the concurrent modulation of sugar metabolism and phenylpropanoid-related pathways. These patterns were supported by the upregulation of GT2-family glycosyltransferase genes and the accumulation of lignin-related flavonoid precursors, such as pinobanksin and pinobanksinol. Collectively, these results highlight statistically robust associations between light-regulated cultivation practices and fruit quality traits, providing a molecular framework for optimizing protected cultivation strategies to enhance both the sensory and nutritional attributes of *P. salicina* fruit without compromising yield.

## 1. Introduction

The Chinese plum (*Prunus salicina* L.), a member of the Rosaceae family, is indigenous to Southeastern China and has achieved global cultivation due to its distinctive flavor profile and nutritional richness. This species is particularly valued for its elevated polyphenol content, which confers significant antioxidant and anti-inflammatory properties. Polymeric condensed tannins derived from plum fruit demonstrate remarkable antioxidant activity [1]. Meanwhile, proanthocyanidins extracted from plum branches exhibit potent anti-tumor effects [2]. Ensuring high-quality plum production is paramount for meeting consumer expectations regarding both sensory attributes and nutritional value.

Fruit quality is a critical determinant of consumer preference and market value. Sugar, acid, and pigments are the core components that shape a fruit’s flavor, appearance, and nutritional value. These components are influenced by genetic factors, environmental conditions, and postharvest treatments. Among environmental cues, light serves as a central regulatory role in fruit tree growth and quality development [3]. In addition to its primary function as the energy source for photosynthesis, light also modulates various metabolic processes, including soluble sugar accumulation [4,5]. Consequently, manipulating the light environment has become an important strategy for improving fruit quality and yield [6].

In modern fruit production, different cultivation systems can create distinct light microenvironments, thereby influencing plant physiology and metabolic regulation. Multi-span greenhouses (M), for example, provide enclosed and highly controlled conditions that reduce overall light intensity and UV exposure while stabilizing temperature and humidity. Retractable electric rain shelters (R) offer partial environmental regulation. On sunny days, it is the same as the control, while on rainy days, an additional covering layer is added for rain protection, and the light intensity is also weakened. In contrast, conventional open-field cultivation (CK) exposes plants to natural sunlight and ambient weather fluctuations, providing the highest light intensity and UV levels. These systems primarily alter illumination intensity and ultraviolet (UV) exposure, potentially activating different photoreceptors (e.g., phytochrome and cryptochrome) and subsequently influencing downstream metabolic pathways. However, the molecular mechanisms linking the light conditions under protected cultivation to fruit quality remain poorly understood.

Research on fruit quality plays a crucial role in guiding breeding programs by identifying superior genotypes and developing strategies to enhance fruit quality and identify health-promoting components with market value [7]. Fruit quality is shaped by the interaction of genetic, environmental, and agronomic factors, with recent multi-omics studies providing new insights into the molecular mechanisms underlying fruit development and ripening. Transcriptomic and metabolomic analyses have revealed key genes and regulatory pathways controlling these processes, highlighting the central roles of transcription factors (TFs) and microRNAs (miRNAs) in coordinating metabolic networks. For example, in bananas, *SPL* TFs targeting miR156 modulate the miR528-*MaPPO* module, influencing cold stress tolerance and postharvest quality [8]. In tomatoes, *SlWRKY35* TF enhances nutritional quality by promoting carotenoid biosynthesis [9]. In Chinese plums, fruit color and sugar content constitute critical varietal traits, with carotenoids being intricately linked to pigmentation and sugar metabolism [10,11]. Anthocyanins, another key determinant of plum coloration, are associated with sugar metabolism and ethylene signaling pathways [12,13]. Notably, previous work has elucidated sugar metabolism pathways in plum and demonstrated that visible light can stimulate anthocyanin biosynthesis through malate dehydrogenase activity and ethylene signaling, underscoring the strong coupling between primary and secondary metabolism during fruit ripening.

Metabolic pathways are central to fruit quality trait manifestation, particularly the balance of soluble sugars and organic acids that define fruit sensory properties [14]. Recent advances in omics technologies, including transcriptomics and metabolomics, have revolutionized our understanding of fruit quality by enabling systematic analysis of gene expression and metabolic profiles during development and ripening. Integrative omics approaches in tropical fruits have identified critical genes and pathways underlying quality traits, offering valuable insights for breeding and cultivation strategies.

Plum (*Prunus salicina* L.) has benefited from these advances, with the release of a high-quality chromosome-level genome assembly that provides an essential foundation for genetic and functional studies. Notably, the genome of the low-chill variety ‘San Yue Li’ has revealed molecular mechanisms underlying flower bud dormancy, offering new insights for cultivar improvement [1]. Among these cultivars, ‘Zuili’ (*Prunus salicina* Lindl. 1830), a historically significant and geographically protected cultivar from Taoyuan Village, Zhejiang Province, is notable for its stringent growth requirements. This cultivar shows sensitivity to temperature and humidity, necessitating greenhouse cultivation. To address these challenges, three cultivation methods were employed. By integrating transcriptomic and metabolomic analyses, we aimed to uncover the regulatory networks and metabolic pathways underlying quality formation in ‘Zuili’ plum under these contrasting cultivation conditions.

## 2. Results

### 2.1. Greenhouse Cultivation Enhances Sugar Content Without Compromising Yield

**Figure 1:**
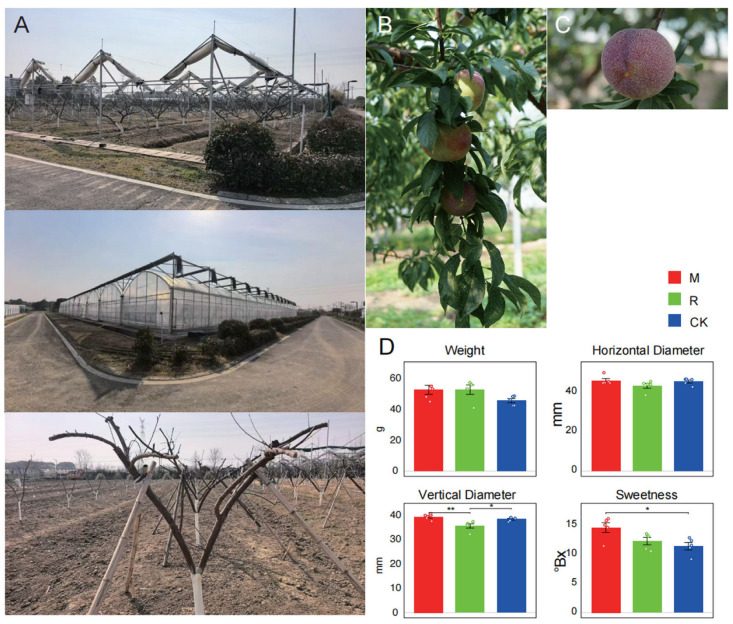
Greenhouse cultivation improves the fruit quality of *Prunus salicina* ‘Zuili’. (**A**) Cultivation methods: retractable electric rain shelter (R), multi-span greenhouse (M), and open field. (**B**,**C**) Morphological images showing fruit size and shape variations. (**D**) Comparative analyses of fruit weight, horizontal diameter, vertical diameter, and sweetness. Each biological replicate corresponds to an independent tree, and fruits harvested from the same tree were pooled prior to measurement to obtain a single value per replicate. Data presented as means ± SD (n = 3). ** *p* < 0.01, * *p* < 0.05. Statistical significance was determined using a one-way analysis of variance (ANOVA).

The *Prunus salicina* ‘Zuili’ plum has high economic value, and in Zhejiang Province, China, while multi-span greenhouse (M) and retractable electric rain shelter (R) systems are commonly used to improve fruit quality and production stability. In this study, we compared the effects of M and R systems on fruit quality and yield, using conventional open-field cultivation (CK) as the control (Figure 1).

Field trials showed mean fruit weights were 52.66 g (M), 52.76 g (R), and 45.60 g (CK), with no significant differences among treatments (*p* > 0.05). Horizontal fruit diameter was also similar across treatments (M: 46.03 mm; R: 43.36 mm; control: 45.52 mm, *p* > 0.05). In contrast, vertical diameter differed significantly: the vertical diameters of fruits grown under M (39.30 mm) were significantly greater than those of fruits grown under CK (38.28 mm, *p* < 0.05) and R (35.55 mm, *p* < 0.01). Meanwhile, R produced fruits with smaller vertical diameters than those grown under CK (*p* < 0.05) (Figure 1D) (File S1).

Soluble sugar content, measured as °Brix, was significantly higher under M (14.68 °Brix) than CK (11.46 °Brix), representing a 28.1% increase (*p* < 0.001). Although R treatment resulted in a moderate increase (12.34 °Brix), the difference was not statistically significant (*p* = 0.12). Importantly, neither M nor R affected yield, indicating that fruit sweetness was improved without compromising productivity.

Pearson correlation analysis revealed significant positive correlation between vertical diameter and sweetness under M treatment (r = 0.72, *p* < 0.05). No such correlation was observed under R or control conditions, highlighting unique physiological effects of M cultivation.

### 2.2. Identification and Functional Enrichment Analysis of Differentially Expressed Genes

**Figure 2:**
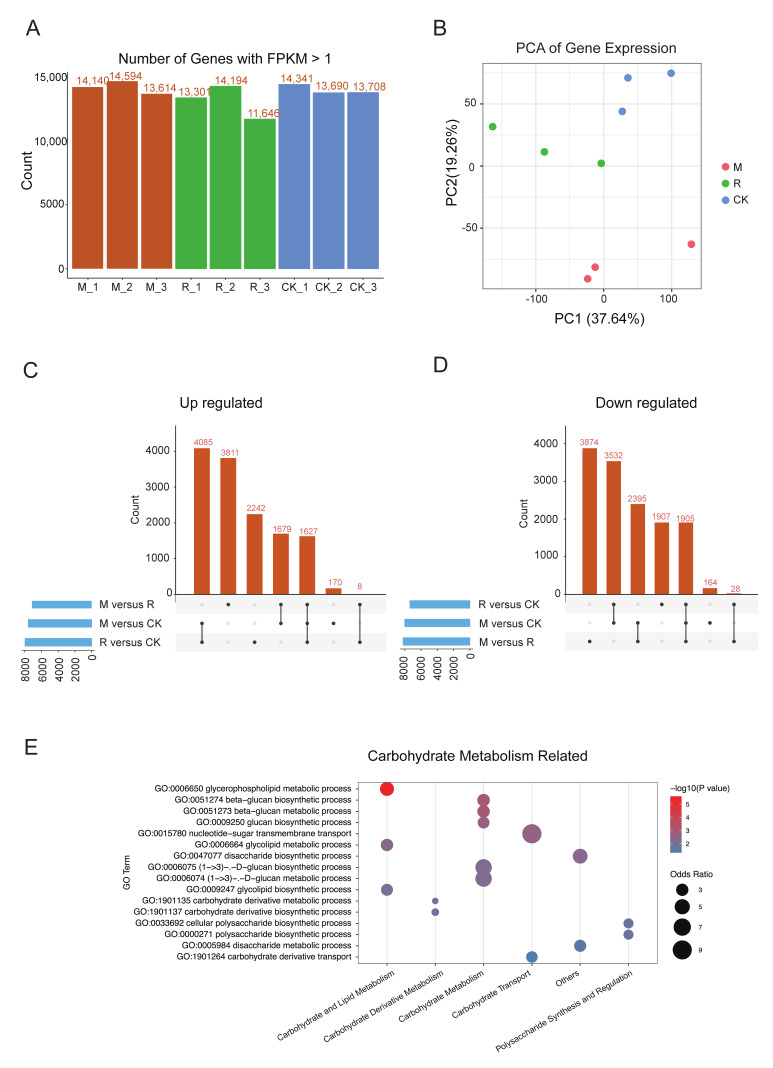
Transcriptional analysis of *Prunus salicina* ‘Zuili’ under different cultivation systems. (**A**) Distribution of expressed genes (FPKM > 1) across samples. (**B**) PCA plot showing treatment group clustering, with PC1 explaining 37.64% and PC2 explaining 19.26% of the variance. (**C**,**D**) Differentially expressed genes: upregulated (**C**) and downregulated (**D**). The bottom panel (dots and connecting lines) indicates the specific intersection of comparison groups, and the top bar chart shows the number of elements in that intersection. (**E**) Enriched carbohydrate metabolism GO terms with gene counts and significance levels. The circle size corresponded to the odds ratio, and the color represented the −log10(*p* value), as shown in the legend.

To elucidate the underlying molecular mechanisms and gene regulatory networks, transcriptome sequencing was conducted at 80 days post-anthesis. Under local field conditions, the fruit development cycle of ‘Zuili’ is approximately 90–100 days from anthesis to maturity, and 80 days post-anthesis corresponds to the post-color-change (veraison) stage. This stage is characterized by rapid sugar accumulation and active metabolic reprogramming, during which differences in fruit coloration and quality traits under contrasting light environments become clearly distinguishable [15]. Three biological replicates per treatment were analyzed, generating a total of 65.66 GB of raw data (average 7.3 GB per sample; File S2). Mapping efficiency ranged from 94.99% to 96.96%, with 14,594–14,647 expressed genes detected per sample (Figure 2A). Principal component analysis (PCA) showed the clear clustering of samples grown under different environments (Figure 2B).

Differential expression analysis revealed that 7561 genes were upregulated in the M treatment and 7962 in the R treatment relative to CK, respectively. Among these, 4085 genes were commonly upregulated under both shading treatments, accounting for 54.0% of M-upregulated genes and 51.3% of R-upregulated gene sets. These shared genes were selected for downstream functional analysis to capture their core molecular responses to shading (Figure 2C,D).

Gene Ontology (GO) enrichment analysis indicated a significant overrepresentation of pathways related to carbohydrate metabolism (Figure 2E). Significantly enriched terms included “disaccharide metabolic process” (GO:0005984), “glycolipid metabolic process” (GO:0006664), “beta-glucan metabolic process” (GO:0051273), and “polysaccharide biosynthetic process” (GO:0000271). Additionally, the term “carbohydrate derivative transport” (GO:1901264) was significantly enriched, suggesting active regulation of sugar transport under shading conditions. Together, these results indicate that shading primarily affects transcriptional regulation of sugar metabolic and transport pathways, which may contribute to the observed increase in fruit sweetness.

### 2.3. Identification and Annotation of Sugar-Related Genes

The concurrent upregulation of genes associated with sucrose metabolism, light signaling, and hormone regulation in both shading treatments provides a molecular basis for the observed increase in fruit sweetness, underscoring the role of shading-induced metabolic reprogramming in enhancing plum fruit quality.

Under the R treatment, multiple genes-encoding sugar-metabolizing enzymes were significantly upregulated compared with those under the CK treatment. These included members of the Glycosyl Hydrolase 36 family (PsSY0005993.1), Glycosyltransferase 2-like (PsSY0001680.1), Plant Galacturonosyltransferase GAUT (PsSY0001680.1), and four Glycoside Hydrolase Family 28 members (PsSY0007071.1, PsSY0008062.1, PsSY0008490.1, PsSY0011831.1). In addition, genes encoding sugar transporters in the major facilitator superfamily (MFS) were upregulated, including PsSY0006090.1, PsSY0006995.1, and PsSY0009625.1, all of which are implicated in transmembrane sugar transport. Several kinase genes involved in sugar metabolic regulation, including AGC kinases, leucine-rich repeat kinases (*PsSY0003101.1*, *PsSY0008284.1*, *PsSY0009894.1*), and S-receptor-like serine/threonine-protein kinase (*PsSY0007795.1*), were also activated, suggesting signaling-mediated transcriptional regulation of sugar metabolism under shading.

Under the M treatment, glycosyltransferase-related genes exhibited upregulation compared to CK. Notably, the phenylpropanoid metabolism pathway underwent substantial restructuring, with secondary metabolites such as vanillin and coniferyl alcohol accumulating at higher levels in M-treated fruits. These compounds may serve as carbon sinks, diverting metabolic flux from primary carbohydrate metabolism toward secondary metabolite biosynthesis while preserving soluble sugar stability, thereby improving fruit flavor and quality.

Within the KEGG phenylpropanoid biosynthesis pathway (ko00940), GT2 (K13065) was identified as a key upregulated gene compared to CK. This glycosyltransferase catalyzes the addition of sugar residues to phenolic compounds, modulating their water solubility and physiological activity. KEGG enrichment analysis further confirmed phenylpropanoid biosynthesis and amino sugar metabolism as top-ranking pathways, indicating coordinated regulation that links enhanced sugar metabolism to the accumulation of phenylpropanoid-derived compounds under shading conditions.

### 2.4. Metabolomic Analysis of Fruit Responses to Shading Conditions

**Figure 3:**
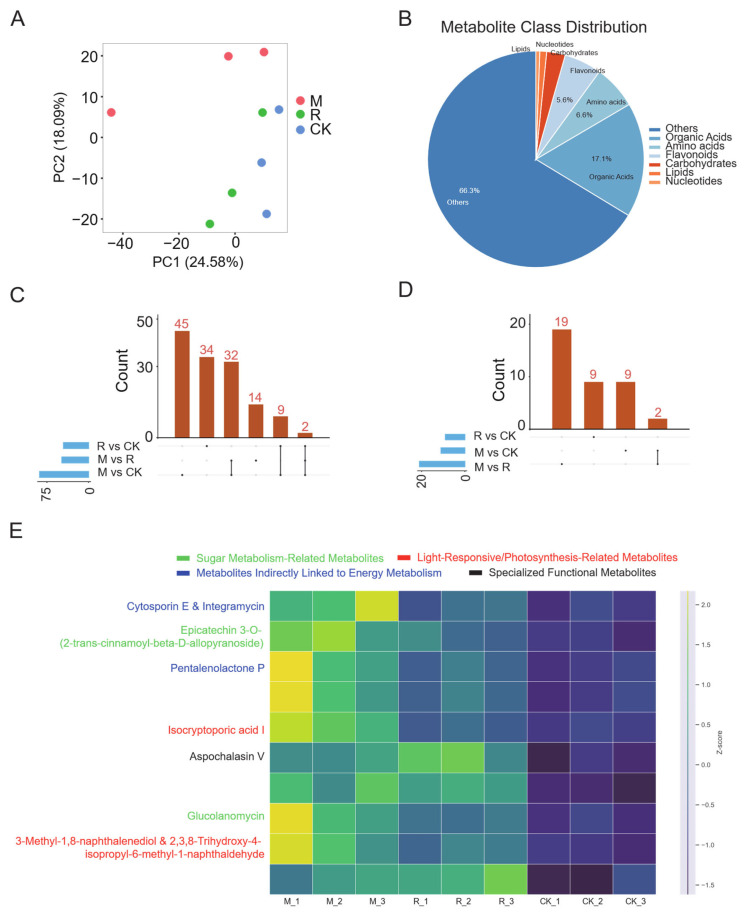
Metabolomic profiling of fruits grown under shading treatments. (**A**) PCA score plot showing group clustering (PC1 = 24.58%, PC2 = 18.09%). (**B**) Metabolite class distribution: organic acids (17.1%), amino acids and derivatives (6.6%), flavonoids (5.6%), carbohydrates (2.8%), lipids (1.0%), nucleotides (0.6%), and others (66.3%). (**C**,**D**) Up- and downregulated metabolites in pairwise comparisons; the bottom panels (dots and lines) visualize the intersection of metabolite sets between treatments. (**E**) Heatmap of key metabolite relative abundance across groups showing treatment-specific signatures.

Metabolomics, which is used to interrogate the repertoire of small molecules within biological systems, provides critical insights into the chemical determinants of fruit flavor. In this study, untargeted metabolomic profiling, following rigorous quality control, identified 1373 metabolites across all samples (Files S3–S5). Principal component analysis (PCA) revealed distinct clustering patterns among treatments (Figure 3A), consistent with the separation observed in transcriptomic data.The complete dataset of all identified metabolites, including their relative abundances and annotation details, is provided in File S6.

Comparative analyses identified 54 differential metabolites (45 upregulated and 9 downregulated) in M versus CK, and 56 differential metabolites (46 upregulated and 10 downregulated) in R versus CK (Figure 3C,D). Eleven metabolites were commonly upregulated under both shading treatments, suggesting shared metabolic responses to reduced light intensity. Among the shared metabolites, epicatechin 3-O-(2-trans-cinamoyl)-β-D-allopyranoside was particularly noteworthy (Figure 3E, Supplementary Materials, Figure S1). This glycosylated flavonoid is implicated in sugar storage and translocation, and its synthesis–hydrolysis dynamics may regulate intracellular free sugar concentrations. The accumulation of this compound under shading conditions is consistent with transcriptomic evidence of enhanced sucrose metabolism, collectively suggesting that shading may stimulate sugar flux through glycoside-mediated pathways.

### 2.5. Correlation Analysis Between Transcriptomics and Metabolomics

**Figure 4:**
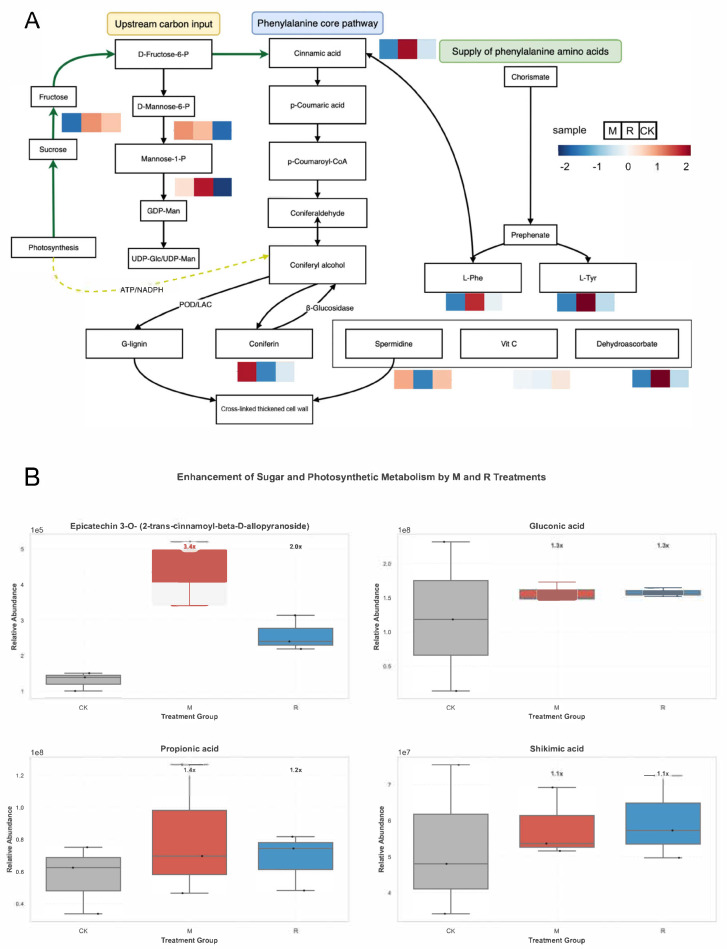
Activation of the phenylpropanoid pathway by M and R treatments. (**A**) Metabolic map of the phenylpropanoid pathway showing changes in metabolite levels for M, R, and CK groups (red: upregulated; blue: downregulated). (**B**) Comparison of four representative metabolites in treated groups compared to the control (CK). One-way analysis of variance (ANOVA) was used for the statistical test.

To elucidate the underlying mechanism for this enhancement, we conducted an in-depth analysis of the phenylpropanoid metabolic pathway (Figure 4A). The pathway map revealed a significant alteration in metabolite accumulation under both treatments, indicating potential adjustments in phenylpropanoid-related pathways. The complete results tables for the GO and KEGG enrichment analyses are provided in Files S7–S9. For instance, the R treatment led to a notable accumulation of key precursor amino acids, L-Phenylalanine (L-Phe), and L-Tyrosine (L-Tyr), which serve as the primary inputs for the entire pathway. Furthermore, the levels of core intermediates, such as Cinnamic acid, were distinctly modulated by the treatments, indicating a targeted regulation of the “Phenylalanine core pathway”. This provides direct evidence at the molecular level that the treatments were actively intervening in this specific metabolic route.

Importantly, changes in upstream-pathway-related processes were accompanied by increased accumulation of several downstream metabolites. We quantified a specific Epicatechin derivative, a major flavonoid compound known for its significant contribution to fruit flavor and antioxidant properties. The results were striking (Figure 4B). Compared to the control (CK), the M treatment induced a highly significant accumulation (*p* < 0.01), with a relative abundance approximately 3.4-fold higher. The R treatment also resulted in a significant (*p* < 0.05) 2.0-fold increase. This demonstrates not only that the pathway was activated, but also that this activation successfully channeled metabolic resources into synthesizing valuable phenolic compounds.

Untargeted metabolomics analysis revealed that both M and R treatments induced significant and specific alterations in major metabolic pathways related to energy and carbon utilization in plum fruits (Figure 4B, Supplementary Materials, Figure S2). A key finding was the directional channeling of resources into secondary metabolic pathways. This was evidenced by the marked accumulation of epicatechin-3-O-(2-trans-cinnamoyl-β-D-glucopyranoside)—a complex phenolic compound—particularly in the M treatment group. This suggests that the treatments created a metabolic state favorable for the synthesis of high-value downstream products. In contrast, the regulation of upstream metabolites in the core metabolic pathways was more intricate. The average levels of gluconic acid and propionic acid—key components of the pentose phosphate pathway—were higher in both the M and R groups compared to the control group. This pattern indicates that these primary metabolites can more rapidly drive other biosynthetic activities.

The connection between primary and secondary metabolism is further supported by the regulation of vanillic acid—a key bridging metabolite derived from photosynthetic precursors. Its content showed a moderate increase under both treatment conditions, indicating sustained or slightly enhanced carbon flow from central metabolism into the aromatic amino acid biosynthesis pathway, which ultimately leads to the production of phenolic compounds.

In summary, this study demonstrates that both M and R treatments induced a metabolically dynamic state in postharvest plum fruits. Rather than simply upregulating all primary metabolic pathways, these treatments appear to have optimized the metabolic network by efficiently redirecting carbon flow from central metabolic pathways—such as the glycolysis and pentose phosphate pathways—toward the synthesis of beneficial secondary metabolites. This targeted reprogramming likely contributed to the improvement of fruit quality.

## 3. Discussion

### 3.1. Application and Value of Protected Horticulture in Fruit Tree Cultivation

The Zuili plum (*Prunus salicina* L. cv. ‘Zuili’), an endangered cultivar endemic to Zhejiang Province, represents not only a valuable horticultural genetic resource for Rosaceae breeding but also an important cultural asset. This landrace variety is characterized by a complex aroma profile dominated by esters and terpenoids, an exceptional sugar–acid balance, and potential stress-resistance traits inherited from its ancestral germplasm [16]. Owing to its high economic and sensory value, the Zuili plum is increasingly cultivated under protected horticultural systems—including greenhouses, plastic tunnels, and controlled-environment facilities.

Our results demonstrate that such protected cultivation systems significantly optimize the growth environment of fruit trees and reduce the impact of extreme climatic events. Regulation of temperature and humidity under protected conditions can manipulation of light intensity, spectrum under facility conditions altered fruit coloration and sugar–acid ratio, indicating that protected horticulture not only stabilizes production output but also improves fruit quality attributes. These findings corroborate highlighting the dual advantage of yield stability and quality enhancement afforded by protected cultivation systems for perennial fruit crops. Previous studies have reported that, while high light intensity generally enhances photosynthetic rates and nutritional quality, excessively strong light can lead to photo-oxidative stress [17]. In contrast, we found that the protected horticultural system, which reduces light intensity, increased fruit sugar content and improved quality in Zuili plums.

### 3.2. Role of Gene Expression Analysis in Understanding Regulatory Mechanisms

Transcriptomic profiling revealed that protected cultivation with shading (M and R treatments) induced substantial transcriptional reprogramming in Zuili plum fruit at 80 days after anthesis. Both shading treatments significantly upregulated genes associated with sucrose metabolism, carbohydrate biosynthetic processes, and glycolysis. These included multiple glycosyl hydrolase family members, glycosyltransferases, and major facilitator superfamily (MFS) sugar transporters. The coordinated upregulation of these genes suggests that shading under protected cultivation promotes both the synthesis and the transport of sugars, forming a molecular basis for increased soluble solids. Under protected greenhouse cultivation, grapes accumulate higher sugar content, which is consistent with the results we have observed with our Zuili plums [18]. Fruit quality is closely related to sucrose regulation, as exemplified by the regulatory network associated with vitamin C and sucrose metabolism in kiwifruit [19].

Furthermore, expression of genes involved in phenylpropanoid metabolism was enhanced, leading to the accumulation of secondary metabolites with potential effects on fruit flavor, texture, and stress tolerance [20,21,22]. The integration of sugar metabolism, phenylpropanoid biosynthesis, and light/hormone signaling pathways under shading conditions indicates a multi-layered regulatory network that simultaneously improves sweetness, aroma, and overall sensory quality. These findings provide direct molecular evidence for the quality-enhancing effects of protected cultivation and highlight potential targets for molecular breeding or precision environmental regulation in fruit production.

### 3.3. Metabolomic Insights into the Mechanism of Quality Improvement

Metabolomic data supported transcriptome findings, revealing that protected shading cultivation coordinates sugar metabolism with phenylpropanoid metabolite accumulation to drive two key quality-promoting molecular routes: (1) sugar accumulation and distribution are enhanced through glycosylation and sugar transport regulation; (2) phenylpropanoid metabolism is boosted, increasing cell wall structural integrity and elevating the content of secondary metabolites with flavor and health-promoting properties. This metabolic reprogramming not only optimizes the nutritional composition of fruits but also improves texture and aroma profiles, without significant yield penalties.

In summary, the integration of transcriptome and metabolome data elucidates how protected cultivation reshapes fruit physiology by aligning carbohydrate metabolism with secondary metabolic pathways through multi-pathway regulation. Such insights underline the high potential of protected horticulture in producing premium-quality fruit, offering a scientific basis for further refinement in the cultivation strategies used for high-value perennial crops.

It should be noted that the regulatory mechanisms proposed in this study are primarily inferred from correlative analyses of transcriptomic and metabolomic data. The integration of multi-omics provides strong associative evidence and valuable hypotheses regarding the molecular basis of quality improvement under protected cultivation; however, the findings remain correlative in nature. Direct functional validation—such as through genetic manipulation (e.g., overexpression or silencing of candidate genes), exogenous application of key metabolites, or controlled environmental experiments targeting specific pathways—was not performed within the scope of this work. Therefore, the proposed regulatory network models, though well-supported by the available data, require further confirmation and refinement through subsequent experimental investigations. Acknowledging this limitation does not diminish the value of the current findings but rather provides a clear and necessary context for their interpretation and highlights important directions for future research.

## 4. Materials and Methods

### 4.1. Experimental Materials

The experiment was conducted at the Sanxin Experimental Base of Tongxiang Agricultural Science Institute in Jiaxing, China (30.6280° N, 120.5640° E). The test materials comprised five-year-old, healthy, uniformly vigorous local plum trees (*Prunus salicina* L.) planted in north–south orientation. Trees exhibited normal growth and fruit production with 3 m × 4 m planting spacing and natural open-center architecture.

### 4.2. Treatment Design

Three cultivation systems were employed to evaluate their effects on fruit quality: Treatment 1 (control, CK)—open-field cultivation under natural conditions; Treatment 2 (multi-span greenhouse, M)—cultivation in a multi-span greenhouse (56 m length × 8 m width × 4.8 m peak height × 3.0 m shoulder height) covered with polyvinyl chloride (PVC) anti-drip dustproof film providing 75% relative light transmittance; Treatment 3 (retractable electric rain shelter, R)—cultivation using a retractable, motorized rain shelter opened during rainy weather and covered with PVC anti-drip dustproof film, offering 70% relative light transmittance.

Fruits were harvested from the same orchard between 9:00 and 10:00 a.m. from identical tree sides and branches. Only intact fruit—free from decay, mold, and mechanical damage—were selected; these were immediately wrapped in aluminum foil, frozen in liquid nitrogen, and stored at −80 °C. Three biological replicates were used for both RNA preparation and metabolite extraction.

Three individual trees were selected as independent biological replicates (n = 3). From each tree, a single fruit was harvested from the southern aspect and middle height of the canopy. Fruits were selected based on consistent maturity (approximately 80 days post-anthesis, at the initial color-break stage). No pooling of fruits was performed; each biological replicate thus consisted of a single fruit from an individual tree to ensure the independence of samples.

### 4.3. Library Preparation and RNA-Seq Sequencing

Total RNA was extracted from plum tissues using TRIzol™ Reagent (Invitrogen, Carlsbad, CA, USA; Cat. No. 15596026) according to the manufacturer’s instructions. RNA purity and concentration were determined on a NanoDrop™ 2000c spectrophotometer (Thermo Fisher Scientific, Waltham, MA, USA; software v1.0.0.66), and RNA integrity was assessed on an Agilent 2100 Bioanalyzer (Agilent Technologies, Santa Clara, CA, USA; RNA 6000 Nano Kit, software B.02.10.SI648), retaining only samples with RIN > 7.0 for library construction. cDNA libraries were prepared using the Illumina TruSeq™ RNA Sample Preparation Kit v2.0 (Illumina, Carlsbad, CA, USA; Cat. No. RS-122-2001).

mRNA was enriched using Dynabeads™ mRNA Purification Kit (Thermo Fisher Scientific, USA; v2.0, Cat. No. 61006) magnetic oligo(dT) beads and fragmented at 94 °C for 8 min in the presence of divalent cations. First-strand cDNA was synthesized with SuperScript™ II Reverse Transcriptase (Invitrogen, USA; v2.0) and random hexamer primers, followed by second-strand synthesis. End repair, A-tailing, and adapter ligation were performed according to the TruSeq protocol, and 300–400 bp fragments were size-selected with AMPure XP beads (Beckman Coulter, Brea, CA, USA). Libraries were PCR-amplified, purified, and evaluated on the Bioanalyzer and by qPCR.

High-throughput sequencing was performed on Illumina NovaSeq 6000 platform generating paired-end reads. Low-quality reads and adapters were removed using fastp (v0.23.2) [23] Clean reads were mapped to the *Prunus salicina* reference genome using HISAT2 (v2.2.1) [24]. Differential expression analysis was conducted using edgeR (version 4.8.1) implemented in R (version 4.4.2). Briefly, raw read counts were imported into edgeR to construct a DGEList object. Genes with low expression were filtered using the filterByExpr() function, which removes genes with insufficient counts across samples based on library sizes and experimental design. Library size normalization was performed using the Trimmed Mean of M-values (TMM) method via the calcNormFactors() function. A design matrix was constructed based on treatment groups, and common, trended, and tagwise dispersions were estimated using the estimateDisp() function. Differential expression testing was conducted using a generalized linear model (GLM) likelihood ratio test, implemented with glmFit() and glmLRT(). The resulting *p*-values were corrected for multiple testing using the Benjamini–Hochberg false discovery rate (FDR) method, as implemented in edgeR. Gene Ontology (GO) analysis was performed using R package GOstats (R Bioconductor package, v2.60.0) [25]. Enrichment significance was evaluated using a hypergeometric test, and multiple-testing correction was performed using the Benjamini–Hochberg false discovery rate (FDR) method. Terms with FDR-adjusted *p*-values below 0.05 were considered significantly enriched.

### 4.4. Untargeted Metabolomic Analysis

Untargeted metabolomic analysis was performed on selected samples. Freeze-dried tissues were pulverized using a mixer mill (MM 400, Retsch, Haan, Germany) with zirconia beads at 30 Hz for 1.5 min. Approximately 100 mg of powdered sample was extracted with 70% (*v*/*v*) aqueous methanol by vortexing and sonication. After centrifugation at 12,000× *g* for 10 min at 4 °C, the supernatants were collected and filtered through 0.22 μm PTFE syringe filters prior to LC–MS analysis.

Chromatographic separation was carried out using an ultra-performance liquid chromatography system (Shim-pack UFLC SHIMADZU CBM30A) coupled to an electrospray ionization tandem mass spectrometer (Applied Biosystems 4500 Q TRAP). Metabolites were separated on an Agilent SB-C18 column (1.8 μm, 2.1 × 100 mm) maintained at 40 °C. The mobile phases consisted of solvent A (water with 0.1% formic acid) and solvent B (acetonitrile with 0.1% formic acid). The flow rate was set to 0.35 mL·min^−1^, and the injection volume was 4 μL. A linear gradient elution was applied as follows: initial 98% A, decreased to 80% A over 10 min, then to 50% A at 13 min, held for 2 min, and finally returned to initial conditions for column re-equilibration.

Mass spectrometric detection was performed on a triple-quadrupole linear ion trap (Q TRAP) mass spectrometer equipped with an electrospray ionization (ESI) source, operating in both positive (ESI^+^) and negative (ESI^−^) ionization modes. The mass scan range was set to *m*/*z* 100–1000. Ion source parameters were as follows: ion spray voltage, +5.5 kV (positive mode) and −4.5 kV (negative mode); source temperature, 550 °C; curtain gas, 25 psi; ion source gas 1 and gas 2, 55 psi. Instrument tuning and mass calibration were performed using polypropylene glycol solutions at concentrations of 10 μmol·L^−1^ and 100 μmol·L^−1^ in QQQ and LIT modes, respectively, to ensure mass accuracy and analytical stability.

Data acquisition was conducted in information-dependent acquisition (IDA) mode, enabling automatic MS/MS fragmentation of the most intense precursor ions for metabolite structural elucidation. Collision energies were applied according to the instrument’s optimized IDA settings.

Raw LC–MS data files were processed using Analyst software (v1.6.3; AB Sciex, Framingham, MA, USA). Peak detection, retention time alignment, and peak integration were performed using a retention time tolerance of 0.2 min and a mass tolerance of 5 ppm. Metabolite annotation was based on accurate mass, retention behavior, and MS/MS spectral matching against public and in-house databases. Identifications were supported by comparisons with reference spectra from mzCloud, MassBank, and KEGG where available. Metabolite abundances were normalized prior to downstream statistical analysis.

Multivariate statistical analyses were performed in R. Principal component analysis (PCA) was conducted using the FactoMineR (version 2.12) and factoextra (version 1.0.7) packages to evaluate global metabolic variation and sample clustering. Orthogonal partial least squares–discriminant analysis (OPLS-DA) was subsequently applied to identify differentially abundant metabolites (DAMs). Metabolites with a variable importance in projection (VIP) value > 1 and an absolute log_2_ fold change (|log_2_FoldChange|) > 1 were considered significantly differentially accumulated (File S10). As this study employed an untargeted metabolomics strategy, metabolite abundances were evaluated on a relative basis rather than absolute quantification.

### 4.5. Statistical Analysis

Three biological replicates were used for all measurements, reported as means ± SD. All statistical analyses were performed using R software (v4.4.1). Prior to hypothesis testing, normality was assessed using the Shapiro–Wilk test, and homogeneity of variances was evaluated using Levene’s test. When the assumptions for parametric analysis were satisfied, one-way analysis of variance (ANOVA) was conducted. Post hoc multiple comparisons were performed using functions implemented in the PMCMRplus package (v1.9.12). A significance threshold of *p* < 0.05 was applied throughout the analysis.

### 4.6. Measurement of Fruit Quality Traits

Fruit weight measurement: Individual fruit weights were measured using an electronic balance. The plums were placed on the weighing pan, and the displayed weight value was recorded. This procedure was repeated five times for each fruit. Fruit size (horizontal and vertical diameter) measurement: A graduated ruler was used for measurement. Both the horizontal and vertical diameters were measured, and the procedure was repeated five times for each fruit. Sugar content (Brix) measurement: A Brix refractometer (Model ST355A) was employed for measurement. The instrument was cleaned and zeroed twice using 0.3 mL of distilled or deionized water. An appropriate amount of liquid sample was then applied, and the measurement button was pressed to obtain the reading. This process was repeated five times.

## Data Availability

The original contributions presented in this study are included in the article. Further inquiries can be directed to the corresponding author. All raw RNA-seq FASTQ files and processed expression matrices have been deposited. The raw data of RNA-seq data are deposited in national genomics data center (NGDC) under the Bioproject PRJCA053073. The raw metabolic data was deposited in figshare: https://doi.org/10.6084/m9.figshare.30909470 (accessed on 22 December 2025).

## Supplementary Materials

The following supporting information can be downloaded at: https://www.mdpi.com/article/10.3390/plants15010164/s1, Supplementary Figure S1. Identification of key differential metabolites using volcano plot analysis. To obtain a global overview of metabolic changes induced by the treatments, a volcano plot analysis was performed. This plot visualizes both the magnitude of change (*x*-axis, log_2_ transformed fold change) and the statistical significance of that change (*y*-axis, -log_10_ transformed FDR q-value) for every detected metabolite. Metabolites that passed the stringent screening criteria (FDR q < 0.05 and fold change > 2 or < 0.5) are highlighted. Specifically, red dots represent metabolites that were significantly up-regulated in the treatment group compared to the control, blue dots represent those that were significantly down-regulated, and gray dots represent metabolites that did not show a statistically significant change. This analysis allowed for the unbiased screening of the most responsive metabolic compounds. Supplementary Figure S2. Functional annotation of differential metabolites via KEGG pathway enrichment. To elucidate the biological functions and systemic roles of the differential metabolites identified in Figure S1, we performed a pathway enrichment analysis based on the Kyoto Encyclopedia of Genes and Genomes (KEGG) database. The results are presented as a bubble chart, where each bubble represents an enriched pathway. The position on the *y*-axis denotes the pathway name, while the position on the *x*-axis (rich factor) indicates the ratio of the number of differential metabolites to the total number of annotated metabolites in that pathway, signifying the level of enrichment. The bubble size corresponds to the count of differential metabolites in the pathway, and the color gradient reflects the *p*-value, with red indicating the highest level of statistical significance. This analysis reveals the key metabolic pathways that were systematically perturbed by the treatments. Supplementary Figure S3. KEGG annotation analysis of differentially expressed genes in the phenylpropane biosynthesis pathway. This figure shows the gene expression regulation of key enzymes in the phenylpropane biosynthesis pathway (KEGG map ko00940). We mapped the differentially expressed genes (DEGs) identified through transcriptomics sequencing to this pathway to visualize the transcriptional level regulation of this metabolic pathway under different treatments. In the figure, each box represents an enzyme catalyzing a specific reaction, identified by its EC number (Enzyme Commission number). The color of the box indicates the expression change of its encoding gene in different comparison groups: The red box indicates that the corresponding gene is significantly upregulated in the R treatment group compared to the control group. The blue box indicates that the corresponding gene is significantly downregulated in the M treatment group compared to the control group. The green box indicates that the corresponding gene is significantly upregulated in both comparison groups. File S1 Key agronomic traits and quality data of Prunus salicina L. fruits under different cultivation modes.xlsx. File S2 Summary of raw sequencing data quality for Prunus salicina fruit samples under different cultivation systems.xlsx. File S3 all metabolite data detected in positive ion mode.xlsx. File S4 all metabolite data detected in negative ion mode.xlsx. File S5 Parameters used in Metabolomics.xlsx. File S6 metabolites across all samples.xlsx. File S7 metabolites kegg annotation.xls. File S8 CK vs. M KEGG.xls. File S9 CK vs. R KEGG.xls. File S10 differentially abundant metabolite.xlsx. Supplementary FigureS1-3.docx.

## References

[1] Yu J., Li W., You B., Yang S., Xian W., Deng Y., Huang W., Yang R. (2021). Phenolic profiles, bioaccessibility and antioxidant activity of plum (*Prunus salicina* Lindl.). Food Res. Int..

[2] Xiang N., Chang X., Qin L., Li K., Wang S., Guo X. (2023). Insights into tissue-specific anthocyanin accumulation in Japanese plum (*Prunus salicina* L.) fruits: A comparative study of three cultivars. Food Chem..

[3] Quian-Ulloa R., Stange C. (2021). Carotenoid Biosynthesis and Plastid Development in Plants: The Role of Light. Int. J. Mol. Sci..

[4] Romanowski A., Monte E., Hernando C.E., Toledo-Ortiz G., Christie J.M., Moreno-Romero J., Halliday K.J. (2024). Editorial: Light-mediated regulation of plant physiology. Front. Plant Sci..

[5] de Wit M., Galvão V.C., Fankhauser C. (2016). Light-Mediated Hormonal Regulation of Plant Growth and Development. Annu. Rev. Plant Biol..

[6] Wu W., Chen L., Liang R., Huang S., Li X., Huang B., Luo H., Zhang M., Wang X., Zhu H. (2024). The role of light in regulating plant growth, development and sugar metabolism: A review. Front. Plant Sci..

[7] Skodra C., Titeli V.S., Michailidis M., Bazakos C., Ganopoulos I., Molassiotis A., Tanou G. (2021). Olive Fruit Development and Ripening: Break on through to the “-Omics” Side. Int. J. Mol. Sci..

[8] Wang H., Wang H. (2015). The miR156/SPL Module, a Regulatory Hub and Versatile Toolbox, Gears up Crops for Enhanced Agronomic Traits. Mol. Plant.

[9] Chinnapandi B., Bucki P., Fitoussi N., Kolomiets M., Borrego E., Miyara S.B. (2019). Tomato *SlWRKY3* acts as a positive regulator for resistance against the root-knot nematode Meloidogyne javanica by activating lipids and hormone-mediated defense-signaling pathways. Plant Signal Behav..

[10] Aono Y., Asikin Y., Wang N., Tieman D., Klee H., Kusano M. (2021). High-Throughput Chlorophyll and Carotenoid Profiling Reveals Positive Associations with Sugar and Apocarotenoid Volatile Content in Fruits of Tomato Varieties in Modern and Wild Accessions. Metabolites.

[11] Vogel J.T., Tieman D.M., Sims C.A., Odabasi A.Z., Clark D.G., Klee H.J. (2010). Carotenoid content impacts flavor acceptability in tomato (*Solanum lycopersicum*). J. Sci. Food Agric..

[12] Chen L., Wang X., Cui L., Li Y., Liang Y., Wang S., Chen Y., Zhou L., Zhang Y., Li F. (2022). Transcriptome and metabolome analyses reveal anthocyanins pathways associated with fruit color changes in plum (*Prunus salicina* Lindl.). PeerJ.

[13] González M., Salazar E., Cabrera S., Olea P., Carrasco B. (2016). Analysis of anthocyanin biosynthesis genes expression profiles in contrasting cultivars of Japanese plum (*Prunus salicina* L.) during fruit development. Gene Expr. Patterns.

[14] Chen T., Zhang Z., Li B., Qin G., Tian S. (2021). Molecular basis for optimizing sugar metabolism and transport during fruit development. Abiotech.

[15] Gou N., Chen C., Huang M., Zhang Y., Bai H., Li H., Wang L., Wuyun T. (2023). Transcriptome and Metabolome Analyses Reveal Sugar and Acid Accumulation during Apricot Fruit Development. Int. J. Mol. Sci..

[16] Solberg S., van Zonneveld M., Diederichsen A. (2024). Editorial: Advances in conservation and utilization of plant genetic resources. Front. Plant Sci..

[17] Gruda N.S., Samuoliene G., Dong J., Li X. (2025). Environmental conditions and nutritional quality of vegetables in protected cultivation. Compr. Rev. Food Sci. Food Saf..

[18] Pinillos V., Ibanez S., Cunha J.M., Hueso J.J., Cuevas J. (2020). Postveraison Deficit Irrigation Effects on Fruit Quality and Yield of “Flame Seedless” Table Grape Cultivated under Greenhouse and Net. Plants.

[19] Han X., Zhang Y., Zhang Q., Ma N., Liu X., Tao W., Lou Z., Zhong C., Deng X.W., Li D. (2023). Two haplotype-resolved, gap-free genome assemblies for Actinidia latifolia and Actinidia chinensis shed light on the regulatory mechanisms of vitamin C and sucrose metabolism in kiwifruit. Mol. Plant.

[20] Xie P., Yang Y., Gong D., Yu L., Wang Y., Li Y., Prusky D., Bi Y. (2024). Preharvest spraying of phenylalanine activates the sucrose and respiratory metabolism in muskmelon wounds during healing. Food Chem..

[21] Chen H., Qiu S., Chen Y., Li J., Xu T., Zhong P., Shao X., Xu S., Ma Z., Huang Z. (2024). Integrated transcriptomics and metabolomics provides insights into the *Nicotiana tabacum* response to heat stress. Front. Plant Sci..

[22] Li C., Wang K., Lei C., Cao S., Huang Y., Ji N., Xu F., Zheng Y. (2021). Alterations in Sucrose and Phenylpropanoid Metabolism Affected by BABA-Primed Defense in Postharvest Grapes and the Associated Transcriptional Mechanism. Mol. Plant Microbe Interact..

[23] Chen S., Zhou Y., Chen Y., Gu J. (2018). fastp: An ultra-fast all-in-one FASTQ preprocessor. Bioinformatics.

[24] Kim D., Paggi J.M., Park C., Bennett C., Salzberg S.L. (2019). Graph-based genome alignment and genotyping with HISAT2 and HISAT-genotype. Nat. Biotechnol..

[25] Falcon S., Gentleman R. (2007). Using GOstats to test gene lists for GO term association. Bioinformatics.

