# Supplementary material for: Multi-Omics Reveals Protected Cultivation Improves Chinese Plum (Prunus salicina L.) Quality via Light-Regulated Sugar Metabolism
Source: Plants (Basel). 2026 Jan 5;15(1):164. doi: 10.3390/plants15010164 (PMC12788113; doi:10.3390/plants15010164)
Supplement: Supplementary file 1 [file plants-15-00164-s001.zip › Supplementary FigureS1-3.pdf]

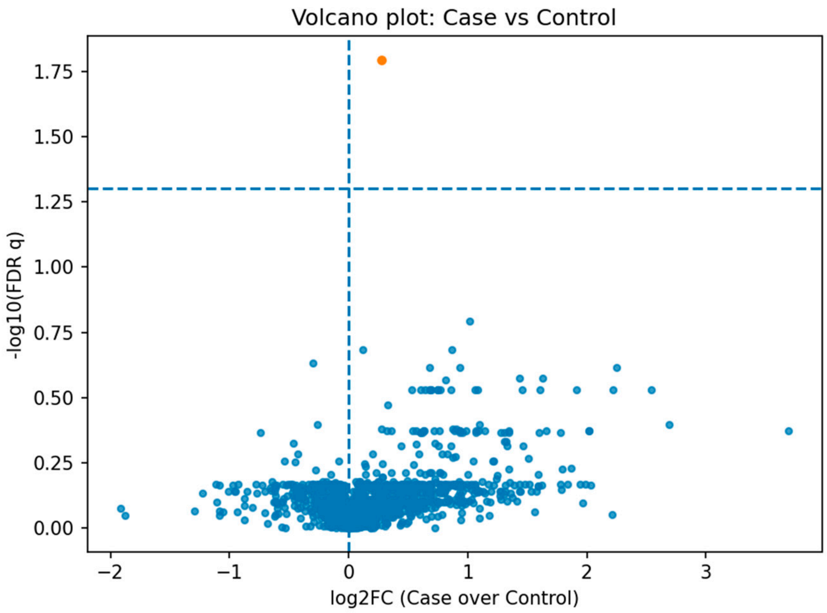

Supplementary Figure S1. Identification of key differential metabolites using volcano plot analysis. To obtain a global overview of metabolic changes induced by the treatments, a volcano plot analysis was performed. This plot visualizes both the magnitude of change (x-axis, $\log_2$ transformed fold change) and the statistical significance of that change (y-axis, $-\log_{10}$ transformed FDR q-value) for every detected metabolite. Metabolites that passed the stringent screening criteria (FDR $q < 0.05$ and fold change > 2 or < 0.5) are highlighted. Specifically, red dots represent metabolites that were significantly up-regulated in the treatment group compared to the control, blue dots represent those that were significantly down-regulated, and gray dots represent metabolites that did not show a statistically significant change. This analysis allowed for the unbiased screening of the most responsive metabolic compounds.

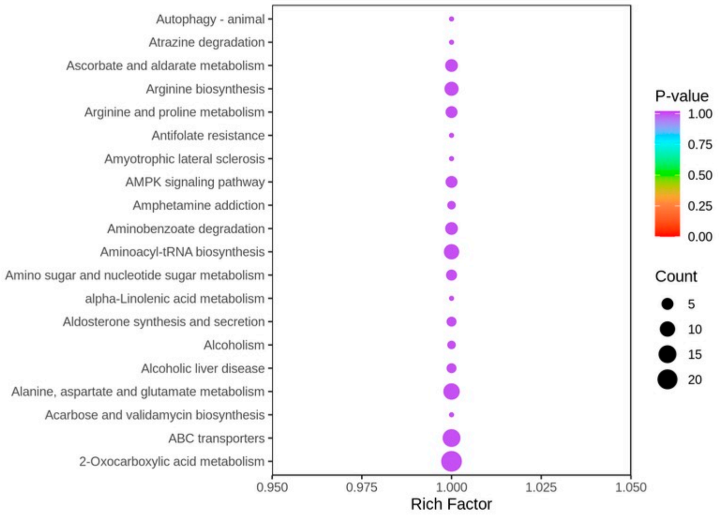

Supplementary Figure S2. Functional annotation of differential metabolites via KEGG pathway enrichment. To elucidate the biological functions and systemic roles of the differential metabolites identified in Figure S1, we performed a pathway enrichment analysis based on the Kyoto Encyclopedia of Genes and Genomes (KEGG) database. The results are presented as a bubble chart, where each bubble represents an enriched pathway. The position on the y-axis denotes the pathway name, while the position on the x-axis (rich factor) indicates the ratio of the number of differential metabolites to the total number of annotated metabolites in that pathway, signifying the level of enrichment. The bubble size corresponds to the count of differential metabolites in the pathway, and the color gradient reflects the P-value, with red indicating the highest level of statistical significance. This analysis reveals the key metabolic pathways that were systematically perturbed by the treatments.

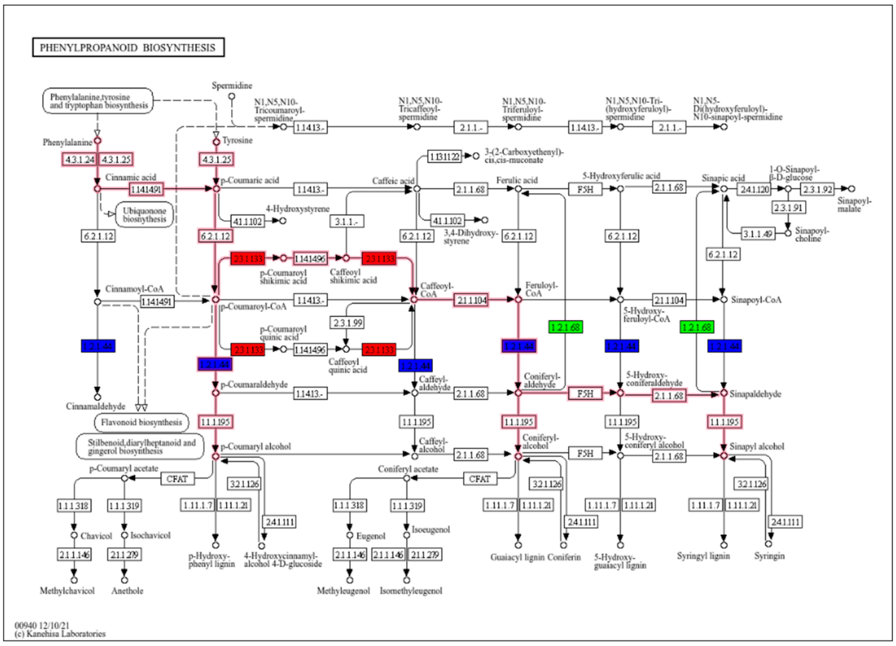

Supplementary Figure S3. KEGG annotation analysis of differentially expressed genes in the phenylpropane biosynthesis pathway. This figure shows the gene expression regulation of key enzymes in the phenylpropane biosynthesis pathway (KEGG map ko00940). We mapped the differentially expressed genes (DEGs) identified through transcriptomics sequencing to this pathway to visualize the transcriptional level regulation of this metabolic pathway under different treatments. In the figure, each box represents an enzyme catalyzing a specific reaction, identified by its EC number (Enzyme Commission number). The color of the box indicates the expression change of its encoding gene in different comparison groups: The red box indicates that the corresponding gene is significantly upregulated in the R treatment group compared to the control group. The blue box indicates that the corresponding gene is significantly downregulated in the M treatment group compared to the control group. The green box indicates that the corresponding gene is significantly upregulated in both comparison groups.
